# Designing Multifunctional Protective PVC Electrospun Fibers with Tunable Properties

**DOI:** 10.3390/polym12092086

**Published:** 2020-09-14

**Authors:** Pedro J. Rivero, Iker Rosagaray, Juan P. Fuertes, José F. Palacio, Rafael J. Rodríguez

**Affiliations:** 1 Engineering Department, Public University of Navarre, Campus Arrosadía S/N, 31006 Pamplona, Spain; rosagaray.105688@e.unavarra.es (I.R.); juanpablo.fuertes@unavarra.es (J.P.F.); rafael.rodriguez@unavarra.es (R.J.R.); 2 Institute for Advanced Materials and Mathematics (INAMAT^2^), Public University of Navarre, Campus Arrosadía S/N, 31006 Pamplona, Spain; 3 Centre of Advanced Surface Engineering, AIN, 31191 Cordovilla, Spain; jfpalacio@ain.es

**Keywords:** PVC, electrospinning, DoE, superhydrophobic, surface roughness, corrosion resistance

## Abstract

In this work, the electrospinning technique is used for the fabrication of electrospun functional fibers with desired properties in order to show a superhydrophobic behavior. With the aim to obtain a coating with the best properties, a design of experiments (DoE) has been performed by controlling several inputs operating parameters, such as applied voltage, flow rate, and precursor polymeric concentration. In this work, the reference substrate to be coated is the aluminum alloy (60661T6), whereas the polymeric precursor is the polyvinyl chloride (PVC) which presents an intrinsic hydrophobic nature. Finally, in order to evaluate the coating morphology for the better performance, the following parameters—such as fiber diameter, surface roughness (Ra, Rq), optical properties, corrosion behavior, and wettability—have been deeply analyzed. To sum up, this is the first time that DoE has been used for the optimization of superhydrophobic or anticorrosive surfaces by using PVC precursor for the prediction of an adequate surface morphology as a function of the input operational parameters derived from electrospinning process with the aim to validate better performance.

## 1. Introduction

Recently, great attention in the scientific community has been paid to the design of engineered wettability surfaces [1] due to their potential applications in a wide variety of industrial applications such as air filtration [2,3], oil/water separation [4,5,6], anti-icing [7,8], antibacterial [9], self-cleaning [10] or even corrosion resistance surfaces [11], among others. Multiple biological surfaces can be found in nature which exhibit a superhydrophobic behavior such as lotus leaves, duck feathers, or legs of the water strider and due to this, the development of bioinspired superhydrophobic surfaces is a continuous challenge [12]. The main strategy is directly influenced by the solid–liquid interfacial energy which has to be minimized by modifying surface chemistry as well as surface roughness. In this sense, the combination of a low surface energy with a hierarchical surface roughness with multiscale surface morphology are the basis to create superhydrophobicity.

Multiple deposition techniques can be found in the bibliography to obtain smart surfaces with highly wetting properties such as chemical vapor deposition [13], laser irradiation [14], lithography [15], layer-by-layer assembly [16], and sol–gel process [17], among others. However, a simple, cost-effective, and highly versatile method for the large-scale fabrication of superhydrophobic nanofibers is the electrospinning [18]. Hydrophobicity or superhydrophobicity are key surface properties for corrosion protection because they result in water and aqueous electrolyte repellence [19] and due to this, multiple research works can be found related to the fabrication of superhydrophobic surfaces for corrosion protection [20,21]. In addition, the use of low-cost and scalable techniques to fabricate superhydrophobic surfaces with multifunctional properties are of great interest for application as robust, self-healing barrier layers [22].

The roughening hydrophobic polymeric materials by nature through electrospinning introduces a highly rough structure as well as low surface energy materials based on nanofibrous mats [23]. In the electrospinning, an electrostatic force is used to obtain electrically charged polymeric jet, which overcomes the surface tension of the polymeric solution. As a result, elongated fibers are accelerated from the capillary tip and deposited onto the collector with corresponding evaporation of the solvent [24,25]. Multiple parameters have to be controlled such as the intrinsic polymeric precursor properties (concentration, molecular weight, viscosity, surface tension, nature of solvent), the operational condition parameters (applied voltage, flow rate, distance of tip to collector), and the environmental conditions (relative humidity, temperature) [26,27]. Another aspect to note is that the surface morphology derived from the electrospun fibers with this specific superhydrophobic behavior can be also implemented for the design of anticorrosive surfaces [28,29]. In this work, polyvinyl chloride (PVC) has been selected for the development of the electrospun fiber mats for being a polymer with a hydrophobic behavior by nature with good anticorrosion properties. The novelty of this work is that, by an adequate control of the input parameters in the electrospinning process such as solution concentration, applied voltage and flow rate makes the fabrication of a desired morphological structure possible. In addition, the use of a design of experiments (DoE) has been conducted in order to define the most representative factors which directly affects to the surface morphology (average fiber diameter, roughness), wettability, and corrosion resistance. The experimental results and the surface responses derived from DoE corroborate that solution concentration has been shown to be the most significant factor in the wettability, enabling the fabrication of hierarchical fiber/bead composites with an intrinsic superhydrophobic behavior, whereas the operational process parameters (applied voltage and flow rate) have shown a much greater effect compared to the solution parameter in the resultant corrosion resistance of the electrospun PVC fibers. Finally, this is the first time that design of experiments (DoE) statistical methodology has been implemented over the electrospinning process of PVC electrospun fibers for the optimization of superhydrophobic or anticorrosive surfaces with the aim to obtain an adequate surface morphology in a certain industrial or engineering application.

## 2. Experimental Section

### 2.1. Materials

Polyvinyl chloride (PVC, *M*_W_ = 80,000 g/mol), dimethylformamide (DMF), and tetrahydrofuran (THF) were purchased from Sigma-Aldrich (Saint Louis, MO, USA) and used without any further purification. Firstly, the fibers obtained by electrospinning were deposited onto standard glass slides for the characterization of the morphological, optical, and wettability properties of the electrospun coatings. Secondly, the electrospun fibers were also deposited onto aluminum substrates of a specific alloy (AA 6061T6) for the characterization of the corrosion tests. Finally, ultrapure water was used in the contact angle measurements of the electrospun fiber surfaces for the wettability tests.

### 2.2. Electrospinning Procedure

Polyvinyl chloride (PVC) has been used as the polymeric precursor for the fabrication of the electrospun fibers. In this work, PVC was dissolved in a 1:1 solvent mixture of THF and DMF in order to obtain a homogeneous PVC solution. In [30], smooth PVC fibers have been obtained from 14 wt % and 16 wt % solutions by using this THF-DMF binary solvent system in a ratio 1:1. However, other works have analyzed the effect of varying THF/DMF volume ratio in the resultant fiber diameter morphology [31,32], showing in all the cases the formation of electrospun fibers in the submicrometric range, although in work [32] it has been found that with the increase of DMF ratio, the fiber diameters became small and the size distribution of the fibers did narrow.

After that, the electrospinning solution was transferred into a 5-mL plastic feeding syringe and by a strict control of both applied voltage and flow rate, the polymeric solution was repelled from the tip to the reference substrate (glass slide or aluminum alloy AA6061) which were fixed onto the aluminum ground collector. A fixed distance of 15 cm between the needle tip and the collector has been performed for all the tests, using a 20-gauge needle with a specific inner diameter of 0.6 mm. All the experiments were conducted at room temperature (20 °C) at 40% relative humidity (RH) and the deposition time was fixed for a specific period of time of 15 min. The electrospinning experiments were conducted at various flow rates and applied voltages by using different PVC concentrations with the aim to fabricate electrospun polymeric fibers with the desired properties in terms of optical transmittance, wettability, surface morphology, and corrosion behavior. Finally, all the electrospun samples have been thermally treated at 80° overnight with the aim to improve the adhesion onto the aluminum reference substrate.

### 2.3. Characterization of the Electrospun Coatings

UV–vis spectroscopy has been used to characterize the optical properties of the electrospun PVC fibers. All optical transmittance spectra were taken in the spectral range from 350 to 900 nm on the UV–vis spectrophotometer at room temperature. A Jasco V-630 spectrophotometer (Agilent, Santa Clara, CA, USA) has been used to perform all the measurements.

Water contact angle (WCA) measurements have been performed by using a contact angle goniometer (CAM 100 KSV Instruments, Burlinghton, VT, USA). The measurements have been performed on five different regions of the electrospun coatings and the average values with their corresponding standard deviation were reported, respectively.

The resultant surface morphologies of the electrospun PVC coatings—which have been obtained by different flow rate, polymer concentration, and applied voltage—have been evaluated by using field emission scanning electron microscope (FE-SEM, Hitachi S4800, Tokyo, Japan). The fiber diameters as well as the surface roughness of PVC fibers were determined by using atomic force microscopy (AFM, Veeco Innova, Plainview, NY, USA). In addition, the adhesion of the coating has been determined by applying a pressure-sensitive adhesive tape onto a grid of small squares previously formed. The fraction of coating removed from the grid when the adhesive tape is removed is compared with standard ratings.

Electrochemical corrosion tests based on Tafel polarization curves were carried out using an Autolab Potentiostat/Galvanostat PGSTAT302 (Metrohm, Herisau, Switzerland). All corrosion tests were conducted at room temperature in 3.5 wt % NaCl by using a conventional three electrode cell system composed of a working electrode (WE, bare or coated aluminum sample), a Ag/AgCl reference electrode (RE), and a platinum counter electrode (CE). It should be noted that before conducting the tests, all the samples of study were exposed in the electrolyte solution for 30 min with the aim to stabilize the open circuit potential (OCP). A linear potential sweep in the anodic direction was conducted at a scan rate of 0.5 mV/s, beginning at 0.15 V below OCP and terminating at 0.15 V above the OCP. The output from these experiments yielded a polarization curve of the current density versus the applied potential. The resulting corrosion current can be calculated by using Tafel slope analysis where it established a relationship between the current density and the electrode potential during the polarization. The corrosion data were obtained from Tafel polarization curves where it was obtained by superimposing a straight line on the linear portions of the cathodic and anodic curves. Finally, other corrosion parameters—such as equivalent weight of the metal, density, or exposed surface—are also required as input parameters. With this information, the software generates the complete set of corrosion parameters. Thus, the corrosion rate is calculated according to

$$\mathrm{Corrosion}\;\mathrm{rate}=327\;\times \;\frac{I_{\mathrm{corr}}.\;M}{V.\;D.\;A}$$

where 327 = 1 year (in seconds)/96,500, and 96,500 = 1 F in coulombs. *I*_corr_ is the corrosion current and is determined by an intersection of the linear portions of the anodic and cathodic sections of the Tafel curves, *M* is the atomic mass, *V* is the valence (number of electrons that are lost during the oxidation reaction), *D* is the density, and *A* is the exposed area of the sample [33].

## 3. Results and Discussion

**Figure 1:**
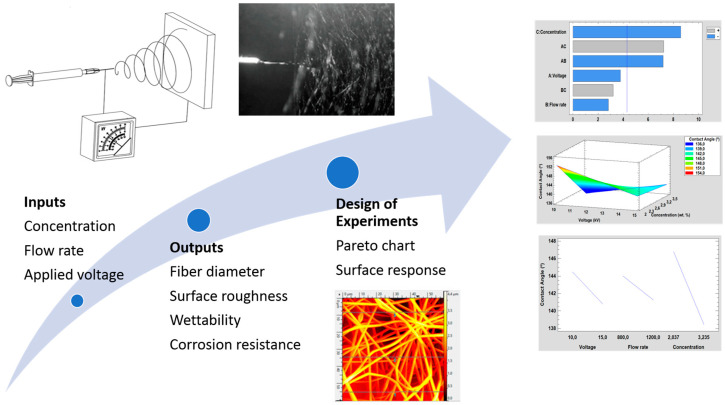
Summary of the operational parameters for the fabrication of the PVC electrospun fibers (polymeric concentration, flow rate, and applied voltage) as well as the output parameters analyzed (fiber diameter, surface roughness, wettability, and corrosion resistance) with their influence by using a design of experiments (DoE).

First of all, it is important to remark that a study about the influence of specific operational parameters—such as polymeric concentration, flow rate, and applied voltage—are evaluated with their corresponding effect in the resultant morphological aspect, optical transmittance, wettability, and the resultant corrosion behavior. In Figure 1, it is shown a summary graph of the input as well as output parameters analyzed in this work. In addition, in Table 1 is presented a summary of the operational parameters for the fabrication of the samples of study. According to this, a total number of eight samples have been fabricated where the PVC concentration was ranged between 10 wt % and 15 wt %, the flow rate was ranged between 800 µL/h and 1200 µL/h and the applied voltage was ranged between 10 KV and 15 KV, respectively.

### 3.1. Surface Morphology

First of all, it is important to remark that the morphological aspect of the electrospun fibers have shown a high degree of variation as a function of the PVC concentration. This difference in the morphological structure of the electrospun fiber mats can be explained by the antagonism between the whipping instability which is caused by the jet to elongate and the surface tension which is responsible for bead formation [34]. This antagonism is mainly affected by the intrinsic polymeric precursor properties, operational parameters, and environmental conditions, and due to this, good control over all these parameters makes possible to tune the fiber diameter and the surface morphology of the electrospun mats [35,36]. Among all them, one of the most relevant parameters in the surface morphology is the polymer concentration because the nanofibers obtained by electrospinning often exhibit beaded fiber structures, which are greatly associated to the solution properties [37]. According to this, when the concentration of the spinning solution is enough high, the resultant viscosity also tends to be high, and as a result, the jet is directly elongated and stretched to the grounded metal collector, enabling the formation of electrospun defect-free fibers [38]. However, when the viscosity of the polymer solution is not high enough to overcome the surface tension, the presence of spindle-like beads is formed during the deposition process [39].

**Figure 2:**
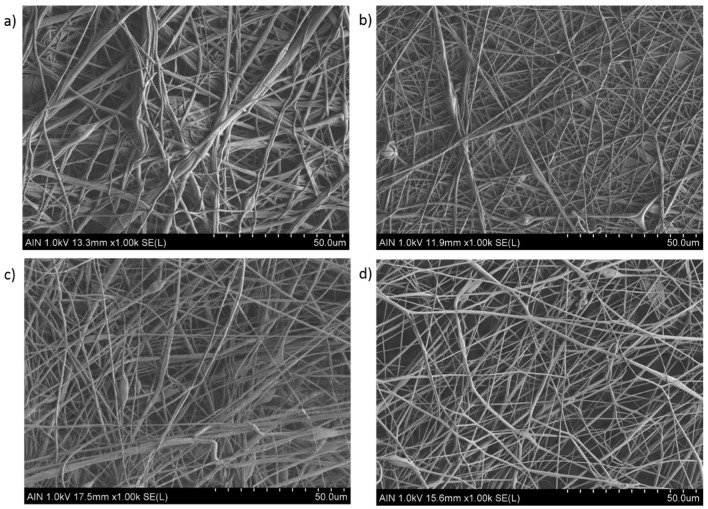
Scanning electron microscopy (SEM) images representing the surface morphologies of the electrospun mats for Sample 1 (**a**), Sample 2 (**b**), Sample 3 (**c**), and Sample 4 (**d**). The scale bar is 50 µm in all images.

**Figure 3:**
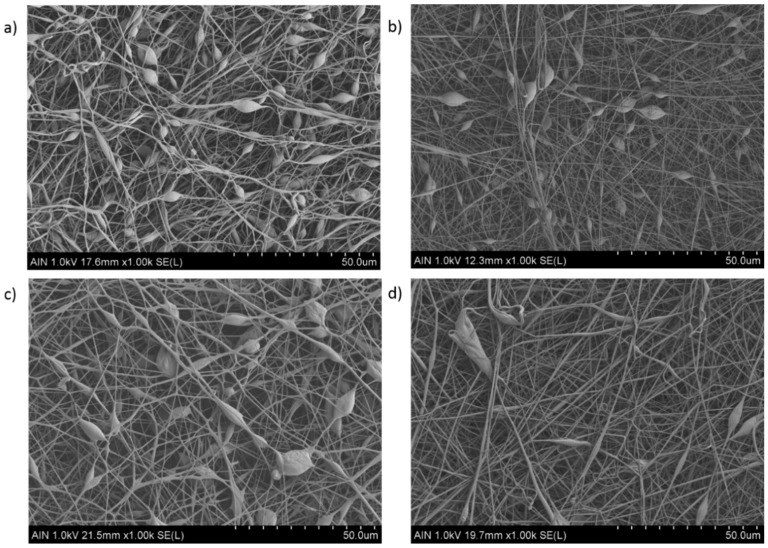
Scanning electron microscopy (SEM) images representing the surface morphologies of the electrospun mats for Sample 5 (**a**), Sample 6 (**b**), Sample 7 (**c**), and Sample 8 (**d**). The scale bar is 50 µm in all images.

In this work, the experimental results obtained by this study for variable PVC concentration are in concordance with literature, as it can be appreciated in the SEM images related to Figure 2 and Figure 3, respectively. In Figure 2, by increasing the PVC solution at a fixed concentration (15 wt %) results in the formation of electrospun fiber mats which are nearly bead-free (Figure 2a–c) or even free (Figure 2d) in comparison with the SEM images in Figure 3 (10 wt %), showing a high density of beaded-fibers. Due to this, it can be confirmed that as a function of the initial polymeric solution, the resultant surface can be perfectly controlled with the desired morphological features. In addition, the effect of applying a higher voltage (15 KV) results in the formation of thinner electrospun fibers (Sample 2 and 4) in comparison with the samples with a lower voltage (10 KV) which corresponds to the Samples 1 and 3, respectively. Previous works have demonstrated that a high voltage allows stretching forces capable of promoting the formation of distributed uniform fibers [40,41] and the effect of increasing the applied voltage produces narrower fibers due to the production of a higher electrostatic repulsive force on the fluid jet [42,43]. Nevertheless, taking into account other works, an adequate selection of the voltage depends on the system used such as type of solvent, polymer concentration, and the distance of the tip to the collector [44,45]. According to the tip-to-collector distance, generally a longer distance yields the formation of thinner electrospun fibers because it is given enough time for stretching of the jet and delayed flight time [46], although it has been also observed that too near or too far distances makes the presence of beads [41] or droplets [47] possible, respectively. However, by using PVC fibers it has been observed that the mean fiber diameter depends little on the change in the distance between the needle and the collector in the range of 7.5 cm up to 15 cm, showing a decrease in the number of fiber defects when the distance is increased [48]. The effect of the flow rate is other parameter that can control the resultant fiber diameter and due to this, low feeding rates are desirable because the solvent has enough time for a complete evaporation before reaching the collector. In addition, high flow rates produce beaded fiber formation due to the unavailability of a proper drying time of the solvent when the collector is reached [49]. In this work, the selected flow rate or applied voltage are not considered too high for being a relevant factor for producing highly-density beaded fibers, as it can be observed in the SEM images of Figure 2.

**Figure 4:**
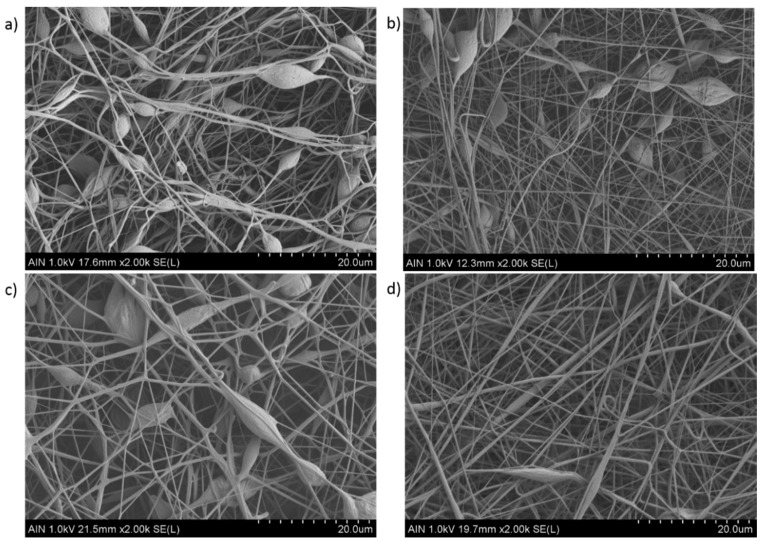
Scanning electron microscopy (SEM) images representing the surface morphologies of the electrospun mats for Sample 5 (**a**), Sample 6 (**b**), Sample 7 (**c**), and Sample 8 (**d**). The scale bar is 20 µm in all images.

As it can be observed in Figure 3, the presence of multiple beads within the fibers are as a result of lower polymer concentration (10 wt %) than critical value and solution viscosity required to maintain a stable polymer jet. This effect is more notorious in the Samples 5, 6, and 7 (Figure 3a–c) than in the Sample 8 (Figure 3d), showing a less amount of beaded fibers. In addition, the magnified SEM images of the PVC mats in Figure 4 clearly show the aspect of the bead-on-string structures with variable sizes and shapes of the beads, being mostly with round or oval shape. After observing all the SEM images can be also concluded that an increase in the fiber diameter has been observed for the highest PVC solution concentration [25], and due to this the average in the fiber diameter is higher in the Samples 1–4 in comparison with the Samples 5–8, respectively. This result is in accordance with the literature because by increasing the solution concentration makes an increase in the resultant fiber diameter possible, preventing the formation of beads within the fibers [50]. Finally, a crosshatch test has performed in order to corroborate the resultant adhesion of the electrospun fibers, showing clearly an increase in the resultant adhesion onto the underlying substrate after thermal treatment. Electrospun samples without thermal treatment were heavily damaged, making it impossible to perform the crosshatch test. However, when the electrospun coating has been thermally treated (80° overnight), the resultant fiber film has been not been destroyed when performing the crosshatch test, although the electrospun coating still showed a low adhesion onto the aluminum substrate because more than 55% of the area was removed when pulling off the tape.

### 3.2. Wettability Properties

**Figure 5:**
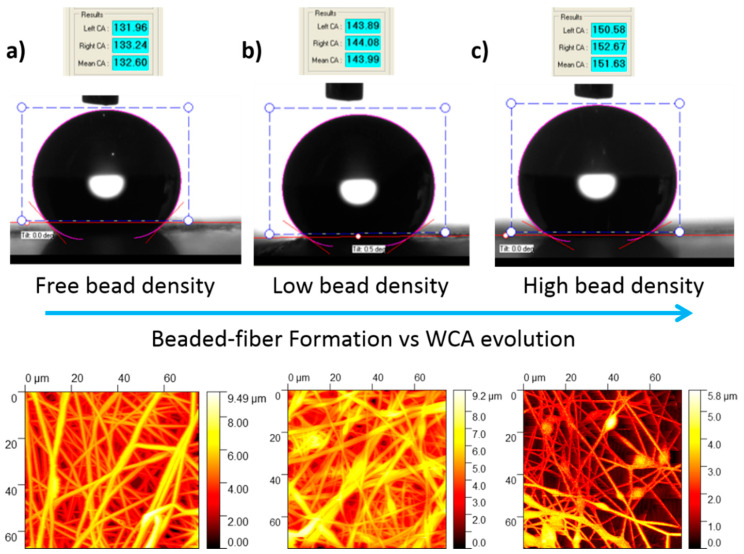
Comparison between the water contact angle images recorded for the PVC electrospun fiber mats with their corresponding AFM images for Sample 4 with free bead density (**a**), Sample 2 with low bead density (**b**), and Sample 5 with high bead density (**c**), respectively.

First of all, it is important to remark that in the present work it has been observed that the water contact angles were high in all the samples of study because of the intrinsic hydrophobic behavior by nature of the PVC associated to the presence of chloride functional groups [51]. However, the resultant WCA measurements on the PVC mats show a wide variation, indicating that electrospun fiber mats with different wettability have been obtained which is associated to the different morphological features, as it was observed in the SEM images. According to this, the minimum WCA obtained was 133.08° (less than 150°) indicating a hydrophobic surface, whereas the maximum WCA value obtained was 151.18° (higher than 150°) indicating that the electrospun fiber mats exhibit superhydrophobic behavior [1]. These high values are attributed to the combination of several factors such as the molecular structure (initial hydrophobicity), the surface roughness of the fibers, the presence of air packets on the fiber film and the bead fiber formation. The experimental results indicate that the maximum WCA value is inherent to the samples with a large beaded fibrous morphology (Sample 5), whereas the minimum WCA value was observed for the sample with beaded-free fibrous morphology (Sample 4). In addition, between both WCA values was observed for the sample with a low bead density (Sample 2). In Figure 5, the WCA values for the samples as well as the surface morphology obtained by AFM images are shown.

The influence of the average roughness and the fiber diameter of the samples has been assessed by AFM and in Table 2 is presented a summary of the experimental data with their corresponding WCA values. First of all, as it was previously commented, the high WCA are associated to the combination of the molecular structure (initial hydrophobicity) and the surface roughness of the fibers. According to this, the improvement in the wettability properties up to superhydrophobic behavior (greater than 150°) of the electrospun fiber mats is mainly caused by the multiscale (hierarchical) roughness of the surface which is induced by the micro/nano-sized fibers combined with the beads [52]. In addition, previous works corroborated that the hydrophobicity increased with a reduction in diameter among bead-free fibers and with the introduction of a high density of relatively small diameters beads [53], even by using other different binary solvents in the formation of the electrospun fibers [54]. The experimental results in this work are in concordance with the literature because Samples 5 and 6 have shown a high density of beads within the fibers with a resultant average fiber diameter in the submicrometric range in comparison with the other samples (micrometric range) and the resultant roughness values are higher for the samples composed of the lowest PVC solution concentration. In addition, the samples with a higher value in the resultant RMS roughness for the highest PVC concentration also shows an increase in the wettability (Samples 2 and 3) which presents low density of beads in comparison with the sample free of beads (Sample 4) or nearly-free beads (Sample 1) which presents a lower value in the roughness. Finally, after comparing these experimental data, it can be corroborated that samples with the presence of beads within the fibers has produced an increase in the WCA values induced by an increase in the corresponding roughness, and as a result, the water droplets sit on the heterogeneous surface of the fiber and the air, resulting in a Cassie–Baxter state [54,55].

### 3.3. Optical Properties

**Figure 6:**
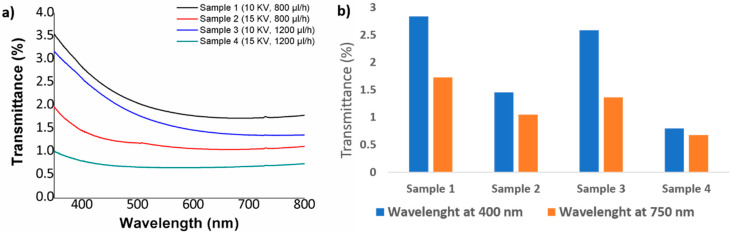
(**a**) UV–vis spectra of the resultant PVC fibers for a fixed polymeric concentration of 15 wt % as a function of the applied voltage (10 or 15 KV) and flow rate (800 or 1200 µL); (**b**) Transmittance change at a specific wavelength of the visible region at 400 nm and 700 nm, respectively.

**Figure 7:**
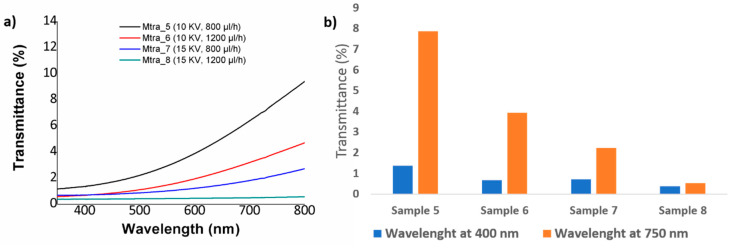
(**a**) UV–vis spectra of the resultant PVC fibers for a fixed polymeric concentration of 10 wt % as a function of the applied voltage (10 or 15 KV) and flow rate (800 or 1200 µL); (**b**) Transmittance change at a specific wavelength of the visible region at 400 nm and 700 nm, respectively.

UV–vis spectra of all PVC samples have been also analyzed in the spectral range of 350–800 nm, as it can be appreciated in Figure 6 and Figure 7, respectively. In both Figures is analyzed the effect of a fixed polymeric concentration (10 wt % and 15 wt %) in the resultant transmittance as a function of variable applied voltage and flow rate. According to this, in the Figure 6 is shown the variation of the transmittance for samples with the highest PVC concentration (15 wt %), and the resultant electrospun PVC fibers have shown a low transmittance and poor transparency in the spectral range of study.

The experimental results indicate that an increase in the flow rate from 800 µL to 1200 µL at a fixed voltage (10 KV or 15 KV) produces a decrease in the light transmittance property. This effect can be clearly appreciated in Figure 2b, with the increase in the flow rate from 800 µL (Sample 1) to 1200 µL (Sample 3) for a fixed voltage of 10 KV, light transmittance property of PVC fibers have decreased from 2.83% to 2.58% and 1.72% to 1.05% for 400 and 700 nm. This same tendency has been observed for the Sample 2 (800 µL/h) and Sample 4 (1200 µL/h) for a fixed voltage of 15 KV where the transmittance was decreased from 1.45% to 0.79% and 1.05% to 0.68% for 400 and 700 nm, respectively. In addition, one aspect to remark is that this effect of reduction in the optical transmittance can be more notorious for an increase in the voltage because if it is compared Sample 1 (10 KV) and Sample 2 (15 KV) for a fixed flow rate at 800 µL/h, the transmittance was decreased from 2.83% to 1.45% and 1.72% to 1.05% for 400 and 700 nm, whereas if it is compared Sample 3 (10 KV) and Sample 4 (15 KV) for a fixed flow rate of 1200 µL/h, the light transmittance property is reduced from 2.58% to 0.79% and 1.35% to 0.68% at the wavelengths of 400 and 700 nm, respectively.

It is worth mentioning that when using a polymeric PVC solution with a low concentration (10 wt %), the electrospun fibers have shown a better transmittance in the spectral range of study (see Figure 7) which is associated to the presence of thinner electrospun fibers in comparison with the fibers obtained by 15 wt % concentration (see Table 2). One aspect to remark is that the same tendency of less transparence is observed when both flow rate or applied voltage are increased. In Figure 7b we can see the evolution from 1.35% to 0.68% at 400 nm and from 7.86% to 3.93% at 700 nm when the flow rate is increased from 800 µL/h (Sample 5) to 1200 µL/h (Sample 6) at a fixed voltage of 10 KV or a change from 0.71% to 0.38% at 400 nm and from 2.2% to 0.52% at 700nm for Sample 7 (800 µL/h) and Sample 8 (1200 µL/h) at a fixed voltage of 15 KV, respectively. In addition, as it has happened for the highest PVC concentration (15 wt %), the effect of increasing the applied voltage from 10 to 15 KV has produced a more significant reduction in the light transmittance of the PVC fibers. More specifically, a change from 1.35% to 0.71% (at 400 nm) and from 7.86% to 2.22% has been observed for Sample 5 (10 KV) and Sample 7 (15 KV) at a fixed flow rate of 800 µL/h or a change from 0.67% to 0.38% (at 400 nm) and from 3.93% to 0.52% for Sample 6 (10 KV) and Sample 8 (15 KV) for a fixed flow rate of 1200 µL/h, respectively. The main conclusion derived from these experimental results are associated to the presence of more air pockets which are trapped between the protrusions on the fiber surface when the flow rate or applied voltage is increased. According to this, these air pockets act as effective pores of the electrospun fiber mats, making a higher scattering of the light possible and as a result, the fiber surface has become more opaque with the corresponding decrease in the optical transmittance in the visible region at 400 nm and 750 nm of the spectral range [56]. It can be concluded as a function of the initial PVC concentration can be manipulated the resultant optical transmittance in the visible region from more opaque (high PVC concentration) up to more transparent (low PVC concentration), although the resultant electrospun fibers show a poor transparency in the visible region. One of the main reasons of this low light transmittance can be derived from the fabrication time of the electrospun fiber coatings and due to this, if the fabrication time is reduced, the fiber surface becomes more transparent. Finally, due to these optical properties of poor transparency, the optical transmittance has not been considered as a representative factor in the design of experiments section.

### 3.4. Anticorrosion Performance

Previous works have demonstrated that the use of electrospun polymeric fibers with an intrinsic hydrophobic behavior by nature onto different metallic substrates (i.e., brass, steel, aluminum, magnesium) have been used for the development of effective anticorrosive surfaces. Some representative examples can be found in the literature such as polyvinylidenefluoride (PVDF) [57,58], polyvinyl chloride (PVC) [59,60], polystyrene (PS) [61,62], core/shell polyacrylonitrile (PAN) fibers reinforced waterborne polyurethane (PU) coating [63], polycaprolactone (PCL) [64,65], functionalized poly(acrylic acid) [66] or block copolymers [28], among others. A summary about different electrospun fibers coatings employed, as well as the parameters used onto the corresponding metallic reference substrate is presented in Table 3.

**Figure 8:**
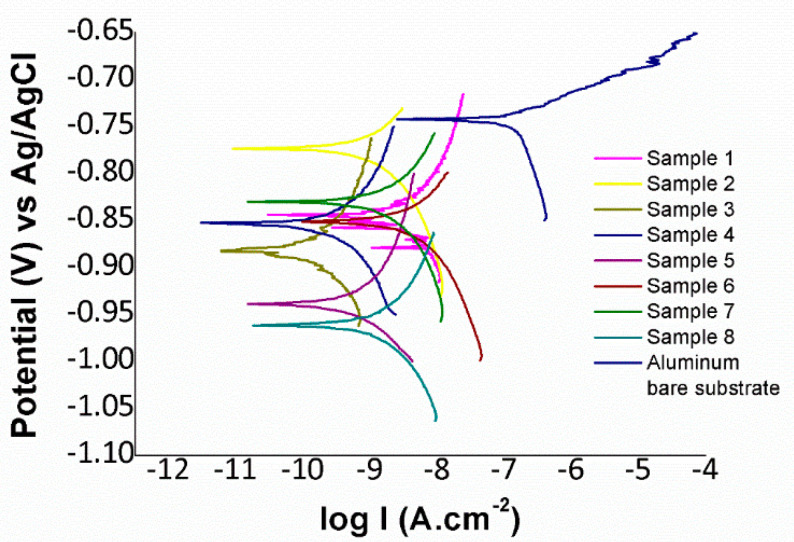
Tafel plots corresponding to the aluminum bare substrate and the different aluminum samples composed of PVC electrospun fiber mats after being tested in 3.5 wt % NaCl aqueous solution.

In order to corroborate that PVC electrospun fibers successfully enhance the corrosion resistance, Tafel polarization tests have been performed in this work. The Tafel plots are displayed in Figure 8 and the results are summarized in Table 4. The main conclusion derived from these results is that all electrospun fiber mat coatings have reduced the corrosion current density and the corrosion rate of the reference aluminum substrate (AA6061T6) in two or even three orders of magnitude, indicating a considerable improvement in the resultant protection efficiency of the reference metallic substrate (AA6061T6). The best results have been found to the Sample 3 which shows the lowest corrosion current density and corrosion rate in comparison with the other samples of study.

### 3.5. Evolution of Raw Data Using Design of Experiments

The technique of design of experiments (DoE) will be used, specifically a central composite design (CCD) with one central point. The factors to be studied (voltage, flow rate and polymer concentration) are shown in Table 5, with their corresponding low and high levels. Likewise, the response variables to be analyzed are the fiber diameter, water contact angle, surface roughness, and corrosion resistance, respectively.

#### 3.5.1. Effect on the Fiber Diameter

**Figure 9:**
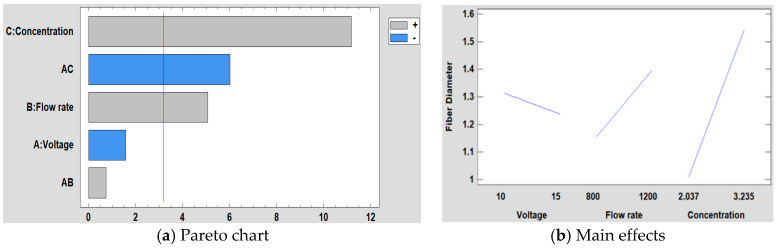
(**a**) Pareto chart and (**b**) the main effects on the average fiber diameter.

In Figure 9 is shown the Pareto chart obtained when performing a statistical analysis of the results for the fiber diameter. It can be stated that the concentration is the most significant factor followed by the interaction between the voltage and concentration, the flow rate, the voltage and the interaction between the voltage and the flow rate. It is shown that when the concentration is increased, the fiber diameter has been also increased as it has been previously commented. In Figure 9b, it is also shown that the effect of the concentration on the fiber diameter is greater than with voltage and flow rate.

The mathematical model for the experimental forces is defined in Equation (1) and the range for each parameter value is determined by the design of the experiments table shown in Table 3. Finally, the adjusted *R*^2^ statistic obtained in the analysis is 95.85%.

Fiber Diameter = −3.0011 + 0.200838 × Voltage + 0.0001625 × Flow rate + 1.63189 × Concentration + 0.000035 × Voltage × Flow rate − 0.0951586 × Voltage × Concentration(1)

**Figure 10:**
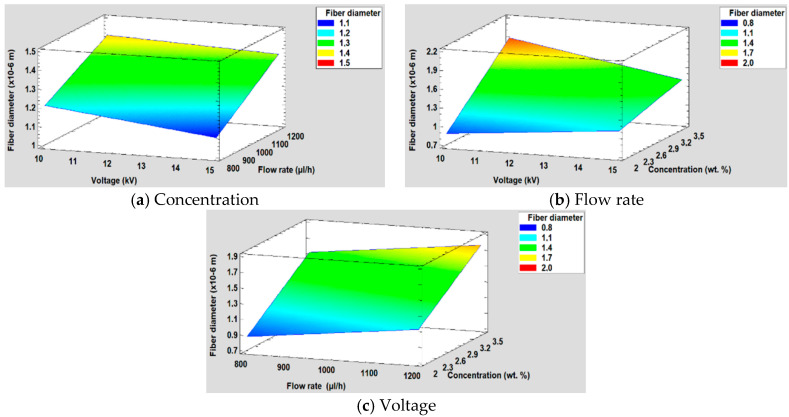
(**a**) Concentration, (**b**) Flow rate and (**c**) Voltage surface response in the average fiber diameter.

Once the Pareto diagram and the mathematical model have been shown, in Figure 10 it is shown the surface response obtained for each factor of study.

#### 3.5.2. Effect on the Surface Roughness

**Figure 11:**
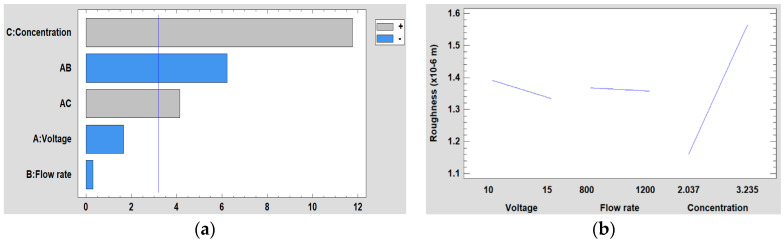
(**a**) Pareto chart and (**b**) the main effects on the surface roughness.

Regarding to the results obtained in the DoE analysis for the surface roughness, it can be seen in Figure 11 that the concentration is the most significant variable, followed by the interaction between voltage and flow rate and the interaction between voltage and concentration. However, it is shown that both the voltage and the flow rate do not have a significant influence, and also, they are the variables with less influence over the resultant surface roughness. This is also corroborated in Figure 11b where it is observed how the line that represents the influence of the concentration has a greater slope than the voltage and the flow rate, respectively. Likewise, it is appreciated that as concentration increases, the surface roughness is also increased.

The *R*^2^ adjusted statistic obtained in the fitting of the model is 99.64% and the equation of the complete model is depicted in Equation (2).

Roughness (× 10^−6^ m) = −0.462528 + 0.0771551 × Voltage + 0.00263825 × Flow rate − 0.254841 × Concentration − 0.000213075 × Voltage*Flow rate + 0.0472705 × Voltage × Concentration (2)

**Figure 12:**
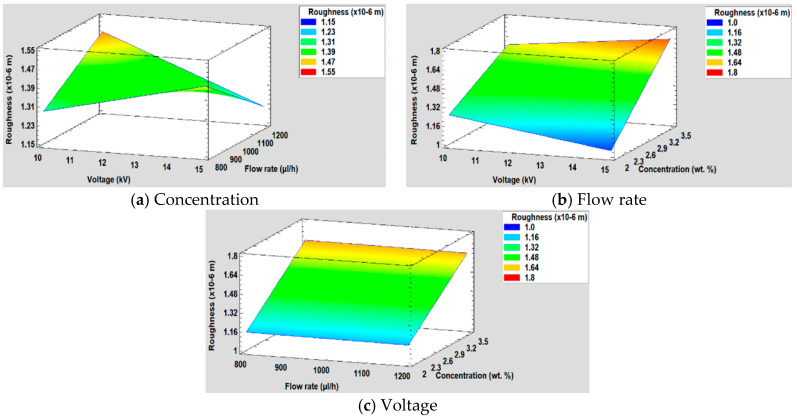
(**a**) Concentration, (**b**) Flow rate and (**c**) Voltage surface response in the surface roughness.

Furthermore, Figure 12 is shown the surface response for the resultant roughness for each of the three factors studied (concentration, flow rate, and voltage).

#### 3.5.3. Effect on the Water Contact Angle

**Figure 13:**
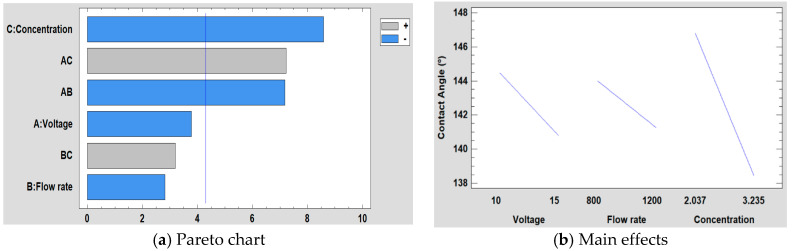
(**a**) Pareto chart and (**b**) the main effects on the water contact angle values.

The study of the influence of concentration, voltage, and flow rate on the water contact angle can be observed in Figure 13. On the one hand, the Pareto chart (Figure 13a) shows that concentration is the most significant factor, followed by the interaction between voltage and concentration and the interaction between voltage and flow rate. On the other hand, in the graph of the main effects (Figure 13b), it is observed how the concentration has more influence on the water contact angle than the flow rate and the voltage. In addition, it is also shown that it has an inverse effect on the contact angle, because when the concentration is higher, the contact angle is smaller. The main reason of this increase in the wettability properties is associated to the hierarchical fiber/bead structure [67], making a multiscale surface morphology possible [68].

**Figure 14:**
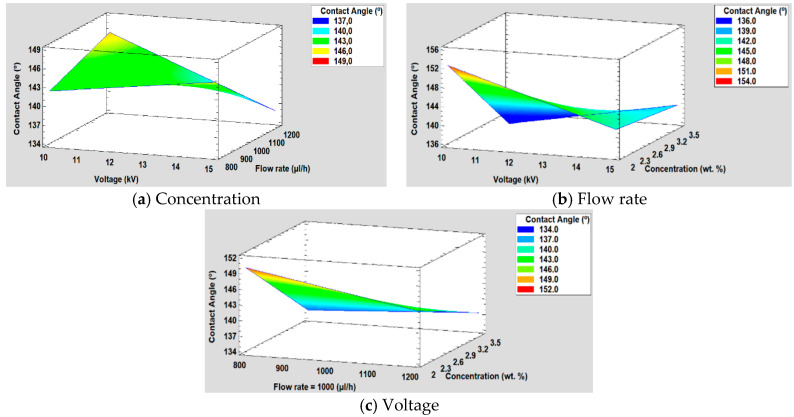
(**a**) Concentration, (**b**) Flow rate and (**c**) Voltage surface response in the water contact angle.

The mathematical model is defined in Equation (3) and, the adjusted *R*^2^ statistic obtained in the analysis is 96.23%. Furthermore, in Figure 14 is shown the surface response for the resultant wettability for each of the three factors studied (concentration, flow rate, and voltage).

Contact Angle (°) = 201.064 + 0.0612654 × Voltage + 0.0459973 × Flow rate − 49.0714 × Concentration − 0.0069525 × Voltage × Flow rate + 2.33639 × Voltage × Concentration + 0.0129278 × Flow rate × Concentration(3)

#### 3.5.4. Corrosion Resistance

**Figure 15:**
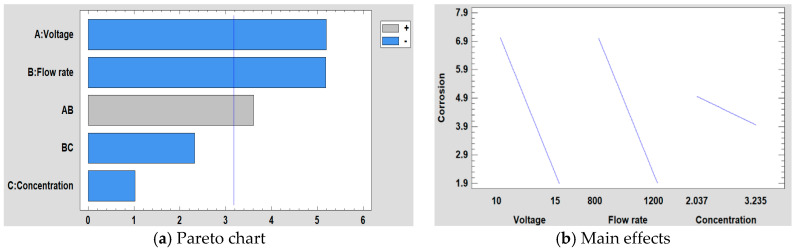
(**a**) Pareto chart and (**b**) the main effects on corrosion resistance.

For the study of corrosion, when performing the DOE analysis, it is observed that in this case, the voltage and the flow rate are the most significant factors followed by the interaction between the voltage and the flow rate (see Figure 15). Likewise, in Figure 15a, it can be seen that both factors have practically the same influence on corrosion, whereas the concentration in this case is not a significant factor, showing a minimal influence. This conclusion is supported by the main effects graph (Figure 15b), where it is observed how the slope of the voltage and the flow rate are practically similar and it is also greater than concentration one. Likewise, it is observed that the variation of voltage and flow rate is inversely proportional to corrosion.

**Figure 16:**
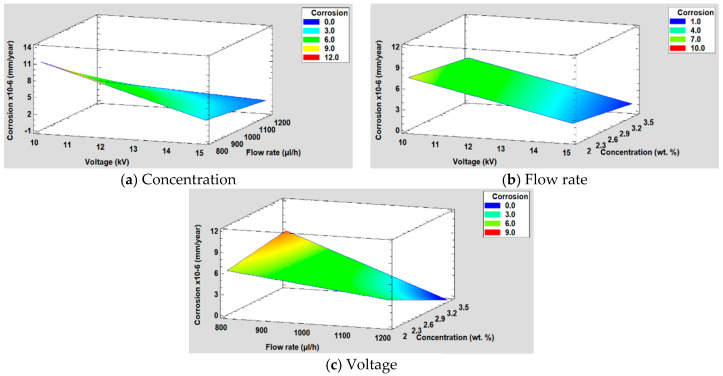
(**a**) Concentration, (**b**) Flow rate and (**c**) Voltage surface response in corrosion resistance.

The equation of the fitted model is shown in Equation (4) and the adjusted *R^2^* statistic obtained in the analysis is 89.52%. Furthermore, the surface response for the corrosion resistance are shown in Figure 16.

Corrosion = 51.3971 − 4.56685 × Voltage − 0.0319732 × Flow rate + 8.67615 × Concentration + 0.00354601 × Voltage × Flow rate − 0.00951328 × Flow rate × Concentration(4)

## 4. Conclusions

In this work, PVC electrospun fiber mats with a desired morphology have been successfully deposited by using the electrospinning technique by controlling three main input parameters such as the polymeric solution concentration, flow rate, and applied voltage. The resultant morphology of the PVC fibers has been evaluated by scanning electron microscopy (SEM) and the average fiber diameter as well as the surface roughness have been assessed by atomic force microscopy (AFM), respectively. A DoE analysis has been carried out where the fiber diameter, surface roughness, water contact angle value, and corrosion resistance have been evaluated as a function of these input parameters. It has been corroborated that the solution parameter was found to be the most influential and significant factor in the surface morphology (i.e., average fiber diameter, roughness) and in the resultant wettability properties. More specifically, the lowest PVC polymeric solution (10 wt %) plays a key factor in the design of a hierarchical fiber/bead surfaces (corroborated by AFM and SEM), showing superhydrophobic behavior with a WCA higher than 150°. However, it has be found that the process parameters (applied voltage and flow rate) have a much greater effect compared to the solution parameter in the resultant corrosion resistance of the electrospun PVC fibers. Finally, the results obtained by this study can serve as a basis for the optimization of both solution and operational parameters, whenever the wettability and/or corrosion resistance are considered as key factors for the design of high-performance applications.

## Figures and Tables

**Table 1:** Summary of the operational inputs parameters for the fabrication of the electrospun fibers as a function of variable polymeric concentration, flow rate, and applied voltage, respectively.

Sample	Concentration (wt %)	Flow Rate (µL/h)	Voltage (KV)
1	15	800	10
2	15	800	15
3	15	1200	10
4	15	1200	15
5	10	800	10
6	10	1200	10
7	10	800	15
8	10	1200	15

**Table 2:** Summary of the WCA values, average fiber diameter, RMS roughness, and mean roughness of all the samples of study.

Sample	Water Contact Angle Measurements (°)	Average Fiber Diameter (µm)	RMS Roughness Sq (µm)	Mean Roughness Sa (µm)
1	133.1 ± 0.8	1.65 ± 0.43	1.19	0.95
2	144.1 ± 4.0	1.18 ± 0.16	1.75	1.43
3	142.1 ± 1.7	1.80 ± 0.61	1.64	1.39
4	134.8 ± 4.2	1.55 ± 0.15	1.36	1.09
5	151.1 ± 2.9	0.78 ± 0.16	1.28	1.06
6	150.6 ± 4.1	1.04 ± 0.15	1.24	1.08
7	145.8 ± 1.8	1.03 ± 0.19	1.04	0.93
8	134.7 ± 4.5	1.21 ± 0.18	0.98	0.87

**Table 3:** Summary of different electrospun coatings with a hydrophobic or even superhydrophobic behavior deposited onto the metallic substrates with their corresponding deposition parameters.

Electrospun Coating	Metallic Substrate	Parameters Used	Ref.
Poly(vinylidene fluoride)/stearic acid (PVDF/SA)	Aluminum sheets	16 KV; 0.8 mL/h; 15 cm distance	[57]
Poly(vinylidene fluoride) (PVDF)	Q345 steel	12 KV; 15 cm distance	[58]
Poly(vinyl chloride) (PVC)	Aluminum, copper, and brass	70 KV; 18 cm distance	[59]
Poly(vinyl chloride)/zinc oxide (PVC-ZnO)	Aluminum alloy (AA6061-T6)	8–14 KV; 0.6–1.2 mL/h; 15 cm distance	[60]
Poly(vinyl chloride)/zinc oxide (PVC-ZnO) and polystyrene/zinc oxide (PS/ZnO)	Aluminum alloy (AA6061-T6)	8–14 KV; 0.6–1.2 mL/h; 15 cm distance for PVC11–17 KV; 1.5 mL/h; 15 cm distance for PS	[61]
Polystyrene/aluminum oxide (PS/Al_2_O_3_)	Commercial aluminum foil	20–25 KV; 1.5–2.0 mL/h; 15 cm distance	[62]
Core-shell polyacrylonitrile fibers reinforced waterborne polyurethane (PU) coating	Hot dip galvanized (HDG) steel	13.5 KV; 13.3/1.0 µL/min; 14 cm distance	[63]
Polycaprolactone/zinc oxide nanoparticles (PCL/ZnO)	Magnesium alloy (AZ31)	16 KV; 1 mL/h; 15 cm distance	[64]
Polycaprolactone (PCL)	Magnesium alloy (AZ31)	200 µL/h; 12 cm distance	[65]
Functionalized poly(acrylic acid) by chemical vapor silanization process	Aluminum alloy (AA6061-T6)	17 KV; 0.5 mL/h; 20 cm distance	[66]
Perfluorinated block copolymer	Aluminum plates	20 KV; 0.8 mL/h; 5–15 cm distance	[28]
Poly(vinyl chloride) (PVC)	Aluminum alloy (AA6061-T6)	Design of experiments (DoE) with 10–15 KV; 0.8–1.2 mL/h; 10–15% solution concentration; 15 cm distance	This work

**Table 4:** Summary table of the Tafel analysis for uncoated aluminum substrate (6061T6) and PVC electrospun coatings of all the samples of this study after being tested in 3.5 wt % NaCl aqueous solution.

Sample	*I*_corr_ (µA/cm^2^)	*E*_corr_ (V)	Corrosion Rate (mm/year)
Aluminum (6061T6)	0.14219	−0.73953	1.4948 × 10^−4^
1	0.0038137	−0.83989	4.0093 × 10^−6^
2	0.0015504	−0.75222	1.6299 × 10^−6^
3	0.0001657	−0.88928	1.7420 × 10^−7^
4	0.00043609	−0.86393	4.5845 × 10^−7^
5	0.00075333	−0.95068	7.9196 × 10^−7^
6	0.005038	−0.83759	5.2963 × 10^−6^
7	0.0023467	−0.82533	2.4670 × 10^−6^
8	0.0017334	−0.96552	1.8223 × 10^−6^

**Table 5:** Design of experiments (DoE) with the factors evaluated with their corresponding low and high level.

Factor	Low Level	High Level
(A) Voltage (kV)	10	15
(B) Flow rate (µL/h)	800	1200
(C) PVC concentration	2.037 g (10 wt %)	3.235 g (15 wt %)
